# Effects of Dietary Bamboo Leaf Flavonoids on Egg Quality, Liver Health, and Inflammatory Responses in Aged Laying Hens

**DOI:** 10.3390/ani16142231

**Published:** 2026-07-18

**Authors:** Xubin Du, Junjun Yuan, Jiawen He, Debing Yu

**Affiliations:** 1 Department of Animal Genetics, Breeding and Reproduction, College of Animal Science and Technology, Nanjing Agricultural University, Nanjing 210095, China; dxb950227@163.com (X.D.); hjw996640759@163.com (J.H.); 2 Joint International Research Laboratory of Animal Health and Food Safety of Ministry of Education, Nanjing Agricultural University, Nanjing 210095, China; 13673552155@163.com; 3 Single Molecule Biochemistry & Biomedicine Laboratory (Sinmolab), Nanjing Agricultural University, Nanjing 210095, China

**Keywords:** bamboo leaf flavonoids, aged laying hens, egg quality, liver health, inflammation, network pharmacology

## Abstract

Bamboo leaf flavonoids (BLFs) possess antioxidant and anti-inflammatory activities; however, their effects in aged laying hens remain unclear. This study investigated the effects of dietary BLF supplementation on production performance, egg quality, and liver health in aged laying hens. The results showed that compared with the control group, BLF significantly improved albumen height, yolk weight, and Haugh units. Histological examination showed that aged laying hens exhibited hepatic steatosis and vacuolar degeneration, whereas BLF supplementation alleviated these pathological changes. In addition, serum levels of aspartate aminotransferase (AST) and alanine aminotransferase (ALT) were significantly reduced in hens fed BLF, indicating that liver cell damage had been alleviated. BLF supplementation increased the hepatic glutathione peroxidase (GSH-Px) and total superoxide dismutase (T-SOD) activities while decreasing the malondialdehyde (MDA) levels, suggesting enhanced antioxidant capacity. Network pharmacology analysis identified major flavonoid components (quercetin, kaempferol, and luteolin) and inflammation-related targets, including IL-6, TNF, and IL-1β. Molecular docking analysis demonstrated strong binding affinities between BLF active components and these key inflammatory targets. Consistently, Western blot analysis showed that BLF reduced the expression of NF-κB, IL-6, and TNF-α in the liver. In conclusion, dietary BLF supplementation improved egg quality, enhanced hepatic antioxidant capacity, and alleviated liver inflammation in aged laying hens. These beneficial effects may be associated with the regulation of inflammatory signaling pathways, highlighting the potential of BLF as a functional feed additive.

## Simple Summary

Aging laying hens often experience reduced egg quality and declining liver health, which can negatively affect poultry production. Natural plant-derived compounds are increasingly being explored as safe nutritional strategies to support animal health and productivity. In this study, we investigated whether bamboo leaf flavonoids, a natural antioxidant extracted from bamboo leaves, could improve egg quality and liver health in aged laying hens. The results showed that dietary supplementation with bamboo leaf flavonoids improved albumen quality, yolk weight, and Haugh units in eggs from older hens. In addition, hens receiving bamboo leaf flavonoids showed reduced liver damage and improved antioxidant capacity. The treatment also decreased inflammatory responses in the liver, suggesting a protective effect against age-related liver dysfunction. These findings indicate that bamboo leaf flavonoids may serve as a promising natural feed additive to maintain liver health and improve egg quality in aging laying hens, providing potential benefits for the poultry industry and animal welfare.

## 1. Introduction

Eggs are a major source of high-quality animal protein, lipids, vitamins, and bioactive compounds in the human diet [1,2]. Egg quality traits, including shell strength, albumen height, yolk composition, and Haugh unit, are therefore critical for both food safety and nutritional value [3,4]. Declines in these traits can lead to economic losses, increased microbial contamination risk, and reduced nutritional and functional quality for consumers. A persistent challenge in commercial egg production is the gradual deterioration of egg quality during the late laying period [5]. As hens age, eggshell strength and thickness decrease, albumen quality deteriorates, and Haugh units decline. Older hens tend to produce heavier eggs with weaker shells and poorer albumen structure, which reduces the economic efficiency of extended laying cycles and decreases the quality of eggs available to consumers.

Liver inflammation has increasingly been recognized as a principal contributor to age-associated egg-quality decline. Recent studies report heightened oxidative stress and inflammatory activation in the livers of older hens, disrupting hepatic homeostasis [6]. These inflammatory alterations impair yolk precursor synthesis and disturb lipid packaging, which compromises yolk quality and subsequently affects albumen structure and eggshell formation. Histopathological findings, including hepatocyte swelling, inflammatory infiltration, and fibrosis-like changes, further correlate with reduced egg quality and diminished laying persistency [7]. Consequently, mitigating hepatic inflammation is considered promising for preserving egg quality during the late laying phase.

Plant-derived flavonoids, widely used in traditional ethnomedicine, have received increasing attention in food and agricultural research due to their antioxidant, anti-inflammatory, and hepatoprotective activities [8]. Dietary flavonoids have been reported to enhance productive performance, improve antioxidant capacity, and alleviate inflammation-associated declines in egg quality [6]. Bamboo leaf flavonoids (BLFs), rich in flavones and flavonoid glycosides, exhibit potent free-radical-scavenging and anti-inflammatory properties and are traditionally valued in East Asian medicine. Although their application in laying hens has been limited, BLFs have shown beneficial effects on antioxidant status and immune regulation in broilers [9], suggesting their potential to protect hepatic function and maintain egg quality in aged layers.

Therefore, this study aimed to investigate the effects of dietary supplementation with BLFs on production performance, egg quality, and liver health in aged laying hens. Using a comprehensive approach combining production performance evaluation, network pharmacology prediction, molecular docking, and histological and biochemical analyses, we explored the potential benefits of BLF in aged hens. We hypothesized that dietary BLF supplementation would improve liver health and antioxidant status, which may contribute to improved egg quality in aged laying hens.

## 2. Materials and Methods

### 2.1. Ethical Approval

The animal study was conducted at the Poultry Experimental Farm of Nanjing Agricultural University in Nanjing, China. All animal experimental procedures and management protocols were strictly reviewed and legally approved by the Institutional Animal Care and Use Committee of Nanjing Agricultural University, Nanjing, China (Approval No: NJAULLSC2025035).

### 2.2. Animals and Experimental Design

A total of 180 Hy-Line Brown laying hens aged 480 days old, with an average initial body weight of 1.92 ± 0.12 kg, were obtained from a commercial laying hen farm (Taizhou, Jiangsu Province, China). Prior to the commencement of the trial, all hens underwent a 2-week acclimation period with ad libitum access to the basal diet and water. Following acclimation, all hens were housed in a fully enclosed lightweight steel-structured poultry house equipped with a longitudinal ventilation system and wet-curtain cooling facilities, and the floor was made of concrete. The hens were randomly allocated to four dietary treatments (3 replicates per group, 15 hens per replicate). Each replicate was maintained in wire cages (length: 50 cm, width: 45 cm, height: 42 cm) equipped with individual nipple drinkers and continuous feed troughs. The birds were housed under strictly controlled environmental conditions, with the ambient temperature maintained at 20–25 °C, relative humidity at 55–65%, and a regulated 16 h light/8 h dark cycle using 30–40 lux white light. During the 42-day experimental period, dietary groups received: basal diet (Con) formulated based on the literature [10], basal diet + 400 mg/kg bamboo leaf flavonoids (L-BLF), Basal diet + 800 mg/kg bamboo leaf flavonoids (M-BLF), and basal diet + 1600 mg/kg bamboo leaf flavonoids (H-BLF). The supplementation levels were selected based on our previous study evaluating bamboo leaf flavonoid supplementation in broilers [11] and the recommended application range of bamboo leaf flavonoids in animal feed. The bamboo leaf flavonoids (effective concentration content ≥ 40%) used in this study were synthesized and produced by Shaanxi Qionghua Biotechnology Co., Ltd., Xi’an, China. Previous studies have identified various flavonoid compounds in bamboo leaf extracts, including orientin, isoorientin, vitexin, isovitexin, quercetin, kaempferol, and luteolin [12]. All hens had free (ad libitum) access to their respective mash feeds and fresh water throughout the entire 42-day experimental period. The replicate cage was considered the experimental unit for production performance measurements, whereas the individual hen was considered the experimental unit for serum biochemical, antioxidant, histopathological, and protein expression analyses. The complete nutritional composition of basal diets is provided in Table 1.

### 2.3. Laying Performance Measurement

Eggs were collected daily during the 42-day experimental period. Egg production, egg weight, and feed intake were systematically recorded to calculate the hen-day egg production and feed conversion ratio (FCR).

### 2.4. Egg Quality Assay

At the end of the feeding trial, eighteen eggs per group were randomly collected and stored at 4 °C for analysis within 24 h. Longitudinal and transverse diameters were measured using a Vernier caliper, with the egg shape index calculated as longitudinal diameter divided by transverse diameter. Eggshell thickness was determined with a spiral micrometer, while eggshell strength was assessed using a KQ-1A eggshell strength tester (Beijing Tianxiang Feiyu Technology Co., Ltd., Beijing, China). Albumen height, Haugh units (HU), yolk color, and yolk weight were measured with an egg quality tester (Toukyo Rhythm Co., Ltd., Tokyo, Japan).

### 2.5. Sample Collection

At the end of the experiment, the hens were fasted for 12 h. Six hens were selected from each treatment group (two from each replicate cage). Approximately 5 mL of blood samples were collected from the wing vein using sterile disposable syringes and transferred into serum collection tubes. The samples were immediately transported to the laboratory for serum separation. After blood collection, the hens were euthanized by cervical exsanguination in accordance with the approved animal welfare protocols. Following confirmation of death, the abdominal cavity was opened under sterile conditions, and the liver was carefully excised. Serum was obtained by centrifugation at 3000 rpm for 15 min and stored at −20 °C until analysis. A portion of the liver was flash-frozen in liquid nitrogen and stored at −80 °C for subsequent biochemical analysis, while another portion was fixed in 4% paraformaldehyde for histopathological examination.

### 2.6. Histopathological Analysis

Liver samples were fixed in 4% paraformaldehyde (4 °C), paraffin-embedded, and sectioned. After deparaffinization, the sections were stained with hematoxylin and eosin (H&E) for histopathological evaluation. The stained sections were subsequently dehydrated, cleared, mounted, and scanned using an Ocus 40 slide scanner (Grundium, Tampere, Finland).

### 2.7. Liver Antioxidant Capacity and Serum Markers of Liver Damage

Liver samples were thawed on ice and homogenized in ice-cold physiological saline (1:9, *w*/*v*) using a tissue homogenizer. The homogenates were centrifuged at 3000 rpm for 10 min at 4 °C, and the resulting supernatants were collected for antioxidant analyses. Serum samples obtained after centrifugation were used for the determination of liver function biomarkers. The activities of catalase (CAT), total superoxide dismutase (T-SOD), glutathione peroxidase (GSH-Px), malondialdehyde (MDA), aspartate aminotransferase (AST), alanine aminotransferase (ALT), and alkaline phosphatase (ALP) were measured using commercial assay kits according to the manufacturer’s instructions.

### 2.8. Screening the Active Ingredients and Targets of BLF

Using the TCMSP database (https://tcmsp-e.com/, accessed on 17 July 2026), the active ingredients of BLF and their target molecules were screened. Oral bioavailability (OB), which reflects the proportion of a compound that can be absorbed and reach systemic circulation, and drug-likeness (DL), which evaluates the structural similarity of a compound to known drugs, were used as screening criteria. Compounds with OB ≥ 30% and DL ≥ 0.18 were selected as candidate active ingredients. The target protein name was converted to the official symbol formats for gene targets using UniProt (http://www.UniProt.org/, accessed on 17 July 2026) databases. The BLF drug–component–target network in was visualized in Cytoscape and the primary active ingredients analyzed based on degree, betweenness centrality (BC), and closeness centrality (CC). Degree reflects the number of connections of a node, BC measures the importance of a node in connecting different parts of the network, and CC reflects how closely a node is connected to all other nodes. Compounds with higher centrality values were considered more likely to play important biological roles [13].

### 2.9. Target Acquisition

To identify potential therapeutic targets for egg quality and liver inflammation in aged laying hens, the GeneCards database (https://www.genecards.org/, accessed on 17 July 2026) was queried using the keyword “Aged laying hens, Egg quality” and “Aged laying hens, Liver inflammation”.

### 2.10. Construction of “Drugs-Active Compounds-Potential Targets” Network

Cytoscape (Version 3.7.2) was employed to establish the “drugs–active compounds–potential targets” network, which included the active compounds, drugs, and potential targets of BLF. In this network, each compound or target is represented by a node, and the relationships between them are depicted as connecting lines.

### 2.11. Drawing of Protein–Protein Interaction (PPI) Networks

Potential targets of BLF against liver inflammation and egg quality were identified via Venny (Version: 2.1.0) (https://bioinfogp.cnb.csic.es/tools/venny/, accessed on 17 July 2026). The PPI network was constructed using the STRING database (https://cn.string-db.org/, accessed on 17 July 2026) with a high-confidence threshold (score > 0.9). Subsequently, Cytoscape (Version: 3.7.2) was employed for network visualization and topological analysis. Key hub targets were identified based on degree, betweenness centrality, and closeness centrality values exceeding the median.

### 2.12. Functional Enrichment Analysis

GO functional annotation and KEGG pathway enrichment analyses were performed using the DAVID database (https://davidbioinformatics.nih.gov/, accessed on 17 July 2026). Significantly enriched terms and pathways were identified based on a threshold of *p* < 0.01. Data visualization was subsequently conducted using the microbiometrics platform.

### 2.13. Molecular Docking Studies

To validate the interactions between BLF components and core targets, molecular docking was conducted using AutoDock Vina (Version: 1.5.7). The 3D structures of target proteins and bioactive ligands were retrieved from the RCSB PDB (http://www.rcsb.org/, accessed on 17 July 2026) and PubChem databases (https://pubchem.ncbi.nlm.nih.gov/, accessed on 17 July 2026), respectively. The PDB IDs of TNF, IL-6, and IL-1β were 2AZ5, 1ALU, and 1ITB, respectively. Ligand files were pre-processed using Open Babel (Version 3.1.1), and PyMOL (https://pymol.org/, accessed on 17 July 2026) was utilized for structural visualization. The docking grid box was defined according to the active binding pocket of each target protein, and the corresponding docking parameters are provided in Supplementary Table S1. Binding affinity was employed as the primary metric to evaluate the potential interactions between ligands and target proteins.

### 2.14. Western Blot Assay

Total protein was extracted with RIPA buffer containing protease inhibitors (Solarbio, Beijing, China), and quantified using a BCA assay (Beyotime, Shanghai, China). Equal protein amounts were resolved on 10% SDS-PAGE (NCM, Suzhou, China) and transferred to nitrocellulose membranes (Merck Millipore, Burlington, MA, USA). Membranes were probed overnight at 4 °C with primary antibodies against NF-κB, TNF-α, IL-6 (1:2000, Proteintech, Wuhan, China), and β-actin (1:2000, Proteintech, China) as a loading control. After TBST washes, HRP-conjugated secondary antibodies (1:5000, Proteintech, Wuhan, China) were applied for 1 h at room temperature. Signals were detected using ECL reagent (Vazyme, Nanjing, China) and quantified via ImageJ (v1.52a).

### 2.15. Statistical Analysis

Data were analyzed using SPSS software (Version 20.0, IBM Corp., Armonk, NY, USA). Prior to analysis, the normality of the data distribution was assessed using the Shapiro–Wilk test, and homogeneity of variances was evaluated using Levene’s test. Data satisfying these assumptions were subjected to one-way analysis of variance (ANOVA). Additionally, orthogonal polynomial contrasts were used to evaluate linear and quadratic dose–response trends. Differences among treatment means were compared using Duncan’s multiple range test. Results are presented as mean ± standard deviation (SD). * Indicates that the difference is significant (*p* < 0.05). ** Indicates that the difference is extremely significant (*p* < 0.01). “ns” denotes non-significant results (*p* > 0.05).

## 3. Results

### 3.1. Effect of BLF Supplementation on the Performance of Laying

**Figure 1:**
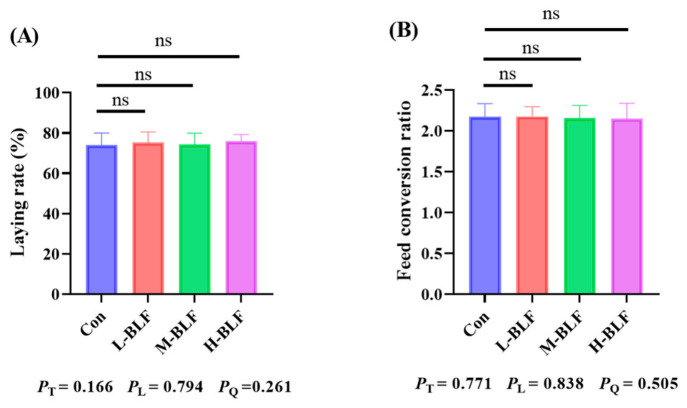
Effects of BLFs on production performance in laying hens. (**A**) Laying rate. (**B**) Feed conversion ratio. Data are expressed as mean ± SD. “ns” denotes non-significant results (*p* > 0.05).

As shown in Figure 1, compared with the Con group, the treatment using BLF did not show any significant effects on the laying rate and feed conversion ratio (*p* > 0.05).

### 3.2. Effect of BLF Supplementation on Egg Quality

**Figure 2:**
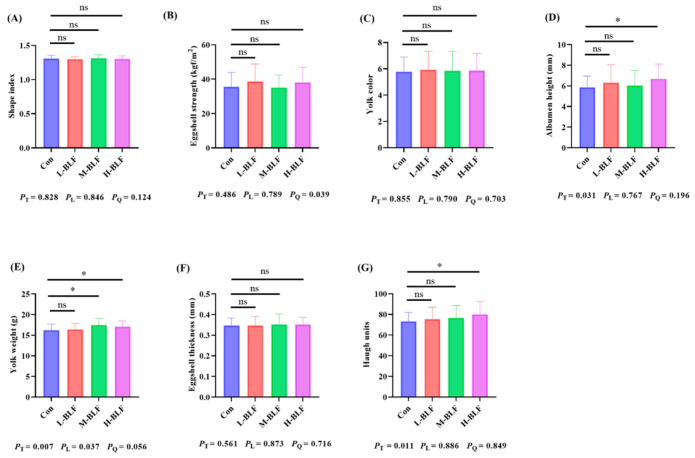
Effects of BLF on egg quality in laying hens. (**A**) Shape index. (**B**) Eggshell strength. (**C**) Yolk color. (**D**) Albumen height. (**E**) Yolk weight. (**F**) Eggshell thickness. (**G**) Haugh units. Data are expressed as mean ± SD. * Indicates that the difference is significant (*p* < 0.05). “ns” denotes non-significant results (*p* > 0.05).

As shown in Figure 2, albumen height was significantly increased in the H-BLF group compared with the Con group (*p* < 0.05). Similarly, yolk weight was significantly higher in the M-BLF and H-BLF groups than in the Con group (*p* < 0.01). Haugh units were significantly increased in the H-BLF group compared with the Con group (*p* < 0.05). No significant differences were observed among groups in shape index, eggshell strength, yolk color, or eggshell thickness (*p* > 0.05). Additionally, orthogonal polynomial contrasts revealed a significant linear trend for yolk weight (*p* = 0.037) and a significant quadratic trend for eggshell strength (*p* = 0.039).

### 3.3. Effects of BLFs on Liver Injury and Oxidative Stress in Aged Laying Hens

**Figure 3:**
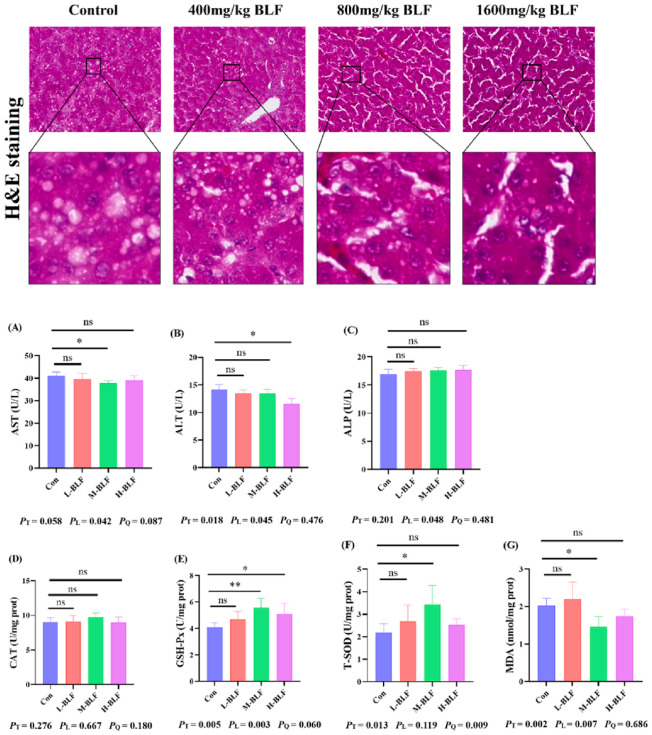
Effects of BLF supplementation on liver health in the aged laying hens. Representative histological images of liver tissues stained with hematoxylin and eosin (H&E) are shown in the upper panels (original magnification ×100), and the corresponding enlarged views are shown below. (**A**) AST, aspartate aminotransferase. (**B**) ALT, alanine aminotransferase. (**C**) ALP, alkaline phosphatase. (**D**) CAT, catalase. (**E**) GSH-Px, glutathione peroxidase. (**F**) T-SOD, total superoxide dismutase. (**G**) MDA, malondialdehyde. Data are expressed as mean ± SD. * Indicates that the difference is significant (*p* < 0.05). ** Indicates that the difference is extremely significant (*p* < 0.01). “ns” denotes non-significant results (*p* > 0.05).

As shown in the Figure 3, aged laying hens exhibited structural damage and vacuolated degeneration in their livers. While vacuolated degeneration was also observed in the livers of hens fed BLFs, the pathological damage was reduced. Furthermore, compared with the control group, the levels of AST (M-BLF) and ALT (H-BLF) in the serum of the BLF group were significantly lower (*p* < 0.05), while no significant difference was observed in ALP (*p* > 0.05). Compared to the Con group, the GSH-Px and T-SOD levels in the liver of M-BLF groups was significantly increased (*p* < 0.05), while the contents of MDA were significantly reduced in the BLF groups (*p* < 0.05). Furthermore, orthogonal polynomial contrasts revealed significant linear and/or quadratic dose–response patterns for these key hepatic damage markers and antioxidant biomarkers.

### 3.4. Network Pharmacology Analysis of BLFs in Relieving Liver Inflammation and Egg Quality Decline

**Figure 4:**
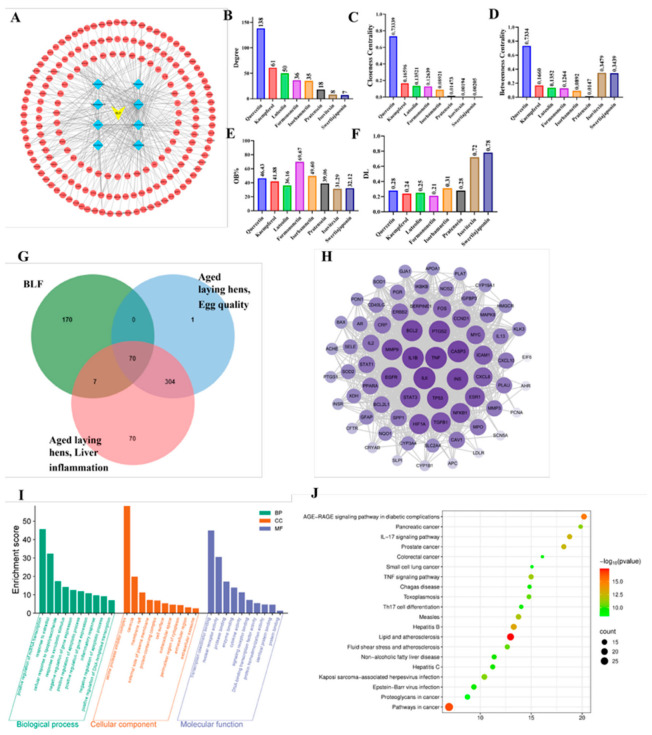
Network pharmacological analysis of BLFs in relieving egg quality decline induced by liver inflammation. (**A**) BLF–primary components–target network diagram showing the interactions between the major active components (quercetin, kaempferol, luteolin, formononetin, isorhamnetin, pratensein, swertiajaponin, and isovitexin) and 143 predicted target genes. Representative target genes include TNF, IL6, NOS2, PTGS2, TLR4, and NFKB1. Red circles represent potential targets of active components. Blue rectangles represent effective active components. Yellow inverted triangles represent BLFs. (**B**) Degree scores of primary components. (**C**) CC scores of primary components. (**D**) BC scores of primary components. (**E**) OB% values of primary components. (**F**) DL values of primary components. (**G**) Target intersection Venn diagram of BLFs, egg quality, and liver inflammation. (**H**) PPI network diagram of intersecting targets. (**I**) GO enrichment analysis of core target genes. (**J**) KEGG pathway enrichment analysis of BLFs for the treatment of liver inflammation.

To elucidate the mechanism of BLFs, active compounds and their corresponding targets were imported into Cytoscape (v3.7.2) to construct a “BLF–component–target” network (Figure 4A–F). Based on the topological analysis of degree, betweenness BC, and CC, six key bioactive components were identified: quercetin, kaempferol, luteolin, formononetin, isorhamnetin, and pratensein. As shown in the Venn diagram (Figure 4G), 70 overlapping targets were identified among the 247 targets of BLFs: 375 targets related to egg quality, and 451 targets associated with liver inflammation in aged hens. Protein–protein interaction (PPI) analysis further revealed that IL-6, TNF, and IL-1β serve as the primary core targets (Figure 4H). GO enrichment analysis (Figure 4I) categorized the top 10 entries in biological processes (BP), molecular functions (MF), and cellular components (CC), highlighting processes such as the inflammatory response, cellular response to lipopolysaccharide, and apoptosis regulation. Furthermore, KEGG pathway analysis (Figure 4J) revealed significant enrichment of the IL-17 and TNF signaling pathways. These findings suggest that inflammatory signaling pathways may be associated with the biological activities of BLF and provide candidate mechanisms for subsequent experimental validation.

### 3.5. Molecular Docking

**Figure 5:**
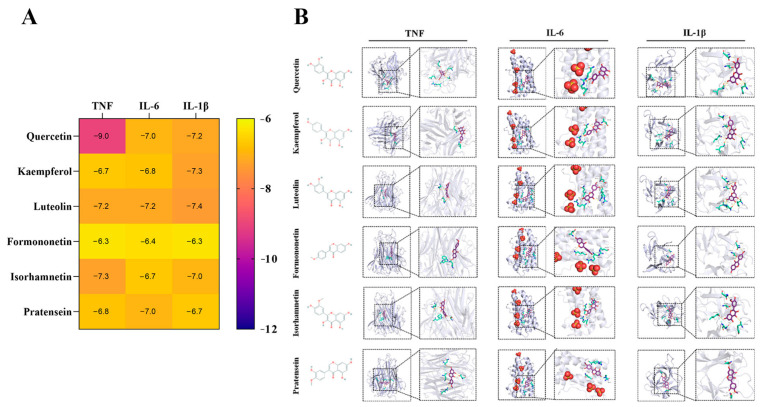
Molecular docking analysis results of BLF core active ingredients with key pro-inflammatory factors. (**A**) Heatmap of binding energies. The color gradient from yellow to purple represents the binding energy from high to low, where a more negative value indicates a more stable ligand-receptor complex. (**B**) 3D visualization of molecular docking. The core components listed are Quercetin, Kaempferol, Luteolin, Formononetin, Isorhamnetin, and Pratensein, which were docked against the target proteins TNF, IL-6, and IL-1β. The purple structure represents the ligand, the cyan sticks represent the key amino acid residues of the receptor, and the yellow dashed lines represent the hydrogen bonds.

To validate the findings from network pharmacology, molecular docking was employed to assess the binding affinity between key compounds and critical targets (Figure 5). The distribution of the docking score was illustrated as heat maps (Figure 5A). It can be seen that all screened compounds exhibited strong binding affinity with the three target proteins, with binding energies ranging from −6.3 to −9.0 kcal/mol. Quercetin exhibited the strongest binding affinity, particularly when interacting with TNF, achieving a minimum binding energy of −9.0 kcal/mol—significantly outperforming other compounds. Luteolin and isorhamnetin exhibited relatively uniform binding energy distributions across the three targets, both remaining stable below −6.7 kcal/mol, indicating the potential for these flavonoids to intervene at multiple targets. Formononetin exhibited relatively higher binding energies (approximately −6.3 to −6.4 kcal/mol) but still demonstrated effective molecular interactions in the docking model. Visualization revealed that within the crystal structures of various core target proteins, the active component of BLFs primarily forms stable bonds with protein receptors through hydrogen bonding, π–alkyl interactions, π–π stacking interactions, and hydrophobic interactions (Figure 5B).

**Figure 6:**
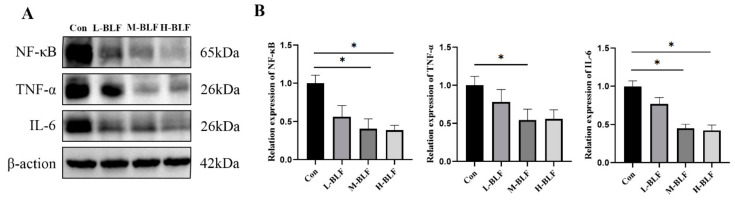
Effects of BLF on the NF-κB pathway. (**A**), Effects of BLF on hepatic NF-κB, TNF-α, and IL-6 protein expression. (**B**), Relative expression levels of proteins. β-actin was used as an internal control for proteins. Data are expressed as mean ± SD. * represents the significant difference compared with the Con group (*p* < 0.05).

To validate the key targets identified by the network pharmacology and PPI analyses, the hepatic protein expression of NF-κB, TNF-α, and IL-6 was evaluated by Western blot analysis (Figure 6). Compared with the Con group, NF-κB protein expression was significantly reduced in the L-BLF and H-BLF groups (*p* < 0.05). Likewise, TNF-α protein expression was significantly reduced in the M-BLF group (*p* < 0.05), whereas IL-6 protein expression was significantly decreased in the L-BLF and H-BLF groups (*p* < 0.05).

## 4. Discussion

Aging in laying hens is characterized by physiological senescence and chronic oxidative stress, which typically culminate in compromised production performance and egg quality. Natural flavonoids have emerged as potent bioactive compounds capable of mitigating age-related functional decline [14]. In the present study, we demonstrated that BLF supplementation effectively improves hepatic health and egg quality in aged laying hens.

The improvement in egg quality, particularly the increases in yolk weight, albumen height, and Haugh unit, suggests that BLF supplementation contributed to maintaining egg quality in aged laying hens. Similar improvements were also observed after adding plant extracts rich in flavonoids to the feed of laying hens [15,16]. These studies, together with the present results, indicate that dietary flavonoids may help alleviate the decline in egg quality associated with aging. Liver function plays an important role in nutrient metabolism and yolk precursor synthesis in laying hens [17,18]. The improved hepatic status observed in BLF-treated hens may therefore be related to the increase in yolk weight. Albumen height and Haugh unit are commonly used indicators of internal egg quality and freshness and generally decline as hens age [19]. Higher values of these parameters are also associated with better processing characteristics and consumer acceptance. The increases observed in the present study indicate that BLF supplementation helped maintain internal egg quality in aged laying hens. Improved antioxidant status has been linked to greater stability of egg quality during storage. The enhanced hepatic antioxidant capacity observed in the present study may therefore have contributed to reducing the quality deterioration. This could be beneficial for maintaining the commercial value of eggs produced by aged laying hens.

The aging process in laying hens is closely associated with oxidative stress, inflammatory damage, and disrupted hepatic lipid metabolism [20]. Dietary supplementation with flavonoids has been demonstrated to alleviate oxidative stress in the body by activating the endogenous antioxidant system [21]. This is consistent with our findings, which demonstrate that the antioxidant effects of BLFs manifest through a significant upregulation of antioxidant enzyme activity in the livers of laying hens. Compared to other plant extracts, BLFs exhibit stronger antioxidant properties, which can be attributed to the high hydrogen-donating efficiency of the phenolic hydroxyl groups (-OH) in their molecular structure, thereby neutralizing ROS and preventing lipid peroxidation [22]. The liver is central to metabolism and stress response; enhancing antioxidant capacity directly alleviates oxidative stress damage to hepatocytes [23]. In our study, while enhancing the antioxidant capacity of the liver, BLFs further improved hepatic health by reducing vacuolar degeneration and decreasing the levels of liver damage markers, specifically AST and ALT. It is therefore inferred that the antioxidant enhancement induced by BLFs synergizes with improved liver health to elevate egg quality in aged laying hens. Given the central role of the liver in nutrient metabolism and yolk precursor synthesis, the improvement in hepatic antioxidant status may contribute to maintaining physiological functions essential for egg formation. This may partially explain the observed improvements in albumen height, yolk weight, and Haugh unit following BLF supplementation. Taken together, the dose–response patterns of egg quality and hepatic antioxidant capacity indicate that 800 mg/kg BLF is the optimal dietary inclusion level for aged laying hens.

Network pharmacology can reveal relationships within drug–target–pathway–disease networks, thereby facilitating the prediction and analysis of drug mechanisms of action, targets, and signaling pathways [24]. Indeed, recent in silico studies have further highlighted the value of such computational approaches in elucidating how natural bioactives modulate the NF-κB pathway to alleviate inflammatory diseases [25]. In preliminary investigation, the core targets we identified were closely associated with inflammatory responses and oxidative stress. In particular, TNF-α, IL-6, and IL-1β exhibited the highest degree values in the PPI network. As core regulators of the inflammatory cascade, they not only play a pivotal role in modulating chronic liver inflammation, but also extensively participate in oxidative stress-mediated tissue damage and subsequent metabolic remodeling processes [26]. Signaling molecules at these key nodes may serve as the primary targets for the pharmacological actions of BLF. Furthermore, evidence suggests that declining egg quality in aged laying hens may be associated with inflammatory responses [27,28].

Research indicates that flavonoids effectively downregulate the levels of TNF-α, IL-6, and IL-1β by inhibiting activation of the NF-κB signaling pathway, thereby alleviating inflammation-induced tissue damage [29]. In this study, molecular docking analysis predicted that BLF components could interact with pro-inflammatory targets, including TNF, IL-6, and IL-1β. Among the identified compounds, quercetin showed the strongest binding affinity with inflammatory proteins. This interaction may be attributed to the hydroxyl-rich (-OH) structure of quercetin, which potentially forms hydrogen bonds with key amino acid residues, including GLN-125 and LEU-37, thereby contributing to the stability of the complex. Additionally, the benzene ring structure of quercetin forms strong π–π stacking interactions with the aromatic amino acid residues of the protein. This geometric complementarity and electrostatic attraction ensure the stability of the complex.

Existing studies have shown that flavonoids can alleviate age-related liver inflammation by suppressing NF-κB signaling and enhancing antioxidant capacity, which is generally consistent with the findings of the present study [30]. In addition, molecular docking analysis suggested strong binding affinities between major BLF components, particularly quercetin and kaempferol, and key inflammatory targets. Combined with the observed reductions in NF-κB, TNF-α, and IL-6 protein expression, these findings suggest that the beneficial effects of BLF on liver health and egg quality may be associated with the modulation of inflammatory responses. Although inflammatory pathways such as TNF and IL-17 signaling have been widely reported in flavonoid research, the present network pharmacology analysis provides a systematic framework linking the major bioactive components of BLF with inflammation-related targets in aged laying hens. Importantly, these computational predictions were further supported by molecular docking and experimental validation, thereby strengthening the evidence for the involvement of inflammatory signaling in the biological effects of BLFs.

It should be noted that the network pharmacology analysis in the present study was based primarily on human gene annotation databases, including TCMSP and GeneCards. Although many inflammatory and oxidative stress pathways are evolutionarily conserved across vertebrates, species-specific differences may influence the target identification and pathway enrichment results. Therefore, further research is needed to validate these interactions and their biological functions at the cellular and molecular levels.

## 5. Conclusions

This study demonstrates that dietary BLFs improve egg quality in aged laying hens, as evidenced by increased albumen height, yolk weight, and Haugh units. BLFs alleviate age-related liver damage and oxidative stress by enhancing antioxidant enzyme activity and reducing the serum levels of liver function biomarkers. Furthermore, BLF supplementation was associated with reduced expression of inflammatory markers, including NF-κB, TNF-α, and IL-6. These effects may collectively contribute to the maintenance of hepatic homeostasis and the improvement of egg quality in aged laying hens. Overall, BLFs show potential as a functional feed additive for aged laying hens.

## Figures and Tables

**Table 1:** Composition and nutrient levels of basal diets (air dry basis, %).

Ingredients	Content
Corn	62.00
Soybean meal	25.00
Limestone	8.00
Premix ^1^	5.00
Total	100.00
Calculated nutrient levels ^2^	
Metabolizable energy (MJ/kg)	11.34
Crude protein	15.52
Lysine	0.71
Methionine	0.36
Methionine + Cysteine	0.53
Threonine	0.66
Calcium	3.40
Available Phosphorus	0.28

^1^ Premix provided the following per kg of the diet: VA 10 000 IU, VD_3_ 3 000 IU, VE 30 IU, VK_3_ 1 mg, VB_1_ 1 mg, VB_2_ 6 mg, VB_6_ 3 mg, VB_12_ 0.01 mg, biotin 0.1 mg, folic acid 0.3 mg, calcium pantothenate 10 mg, nicotinamide 40 mg, choline chloride 350 mg, NaCl 3 g, methionine 1 g, Cu (as copper sulfate) 8 mg, Fe (as ferrous sulfate) 80 mg, Mn (as manganese sulfate) 100 mg, Zn (as zinc sulfate) 60 mg, I (as potassium iodide) l mg, Se (as sodium selenite) 0.3 mg. ^2^ Nutrient levels were calculated values.

## Data Availability

The raw data supporting the conclusions of this article will be made available by the authors on request.

## Supplementary Materials

The following supporting information can be downloaded at: https://www.mdpi.com/article/10.3390/ani16142231/s1, Supplementary Table S1. Docking grid box parameters used for molecular docking analysis between the major bioactive components of bamboo leaf flavonoids (BLF) and inflammatory target proteins.

## Abbreviations

The following abbreviations are used in this manuscript:

ALT	Alanine aminotransferase
ALP	Alkaline phosphatase
AST	Aspartate aminotransferase
BLF	Bamboo leaf flavonoids
CAT	Catalase
GO	Gene Ontology
GSH-Px	Glutathione peroxidase
IL-6	Interleukin-6
KEGG	Kyoto Encyclopedia of Genes and Genomes
MDA	Malondialdehyde
NF-κB	Nuclear Factor Kappa-B
PPI	Protein-Protein Interaction
T-SOD	Total Superoxide Dismutase
TNF-α	Tumor Necrosis Factor-A
HU	Haugh unit
ROS	Reactive oxygen species
