# Supplementary material for: Effects of Dietary Bamboo Leaf Flavonoids on Egg Quality, Liver Health, and Inflammatory Responses in Aged Laying Hens
Source: Animals (Basel). 2026 Jul 18;16(14):2231. doi: 10.3390/ani16142231 (PMC13405878; doi:10.3390/ani16142231)
Supplement: Supplementary file 1 [file animals-16-02231-s001.zip › animals-4385913-supplementary.pdf]

Supplementary Table S1. Docking grid box parameters used for molecular docking analysis between the major bioactive components of bamboo leaf flavonoids (BLF) and inflammatory target proteins.

| **Ligand** | **Target Protein** | **PDB ID** | **Center X** | **Center Y** | **Center Z** | **Size X** | **Size Y** | **Size Z** |
|---|---|---|---|---|---|---|---|---|
| Isorhamnetin | TNF-α | 2AZ5 | 20.083 | 50.979 | 40.936 | 63.000 | 63.000 | 61.500 |
| Formononetin | TNF-α | 2AZ5 | 20.503 | 51.034 | 41.053 | 63.350 | 63.350 | 61.842 |
| Kaempferol | TNF-α | 2AZ5 | 20.698 | 49.892 | 39.738 | 62.611 | 65.333 | 65.333 |
| Luteolin | TNF-α | 2AZ5 | 20.725 | 50.141 | 41.218 | 62.456 | 63.875 | 61.036 |
| Pratensein | TNF-α | 2AZ5 | 20.083 | 50.702 | 41.275 | 63.000 | 63.000 | 61.500 |
| Quercetin | TNF-α | 2AZ5 | 20.529 | 50.365 | 40.130 | 62.144 | 63.467 | 63.467 |
| Formononetin | IL-1β | 1ITB | 22.936 | -1.092 | 75.134 | 44.000 | 45.000 | 40.000 |
| Isorhamnetin | IL-1β | 1ITB | 23.100 | -1.060 | 75.086 | 44.000 | 45.000 | 40.000 |
| Kaempferol | IL-1β | 1ITB | 23.154 | -1.118 | 75.356 | 44.000 | 45.000 | 40.000 |
| Luteolin | IL-1β | 1ITB | 22.821 | -1.090 | 74.966 | 44.000 | 45.000 | 40.000 |
| Pratensein | IL-1β | 1ITB | 22.986 | -1.287 | 74.963 | 44.000 | 45.000 | 40.000 |
| Quercetin | IL-1β | 1ITB | 22.930 | -1.006 | 74.798 | 44.000 | 45.000 | 40.000 |
| Formononetin | IL-6 | 1ALU | 0.248 | -20.016 | 8.749 | 45.000 | 50.000 | 42.000 |
| Isorhamnetin | IL-6 | 1ALU | 0.196 | -19.736 | 9.253 | 46.000 | 50.000 | 40.000 |
| Kaempferol | IL-6 | 1ALU | 0.248 | -20.016 | 8.749 | 45.000 | 50.000 | 42.000 |
| Luteolin | IL-6 | 1ALU | 0.248 | -20.016 | 8.749 | 45.000 | 50.000 | 42.000 |
| Pratensein | IL-6 | 1ALU | 0.248 | -20.016 | 8.749 | 45.000 | 50.000 | 42.000 |
| Quercetin | IL-6 | 1ALU | 0.248 | -20.016 | 8.749 | 45.000 | 50.000 | 42.000 |

Note: Molecular docking was performed using AutoDock Vina. The docking grid box was defined according to the active binding pocket of each target protein. Binding affinity values (kcal/mol) were used as indicators of the predicted binding potential between ligands and target proteins.
